# Supplementary material for: Modeling glioblastoma heterogeneity as a dynamic network of cell states
Source: Mol Syst Biol. 2021 Sep 16;17(9):e10105. doi: 10.15252/msb.202010105 (PMC8444284; doi:10.15252/msb.202010105)
Supplement: Supplementary file 6 — Source Data for Figure 5 [file MSB-17-e10105-s004.zip › Figure5A_sourcedata/GSEA_3017/nosigdb_stateB.GseaPreranked.1622795021725/DOROSZKO_U3054MG_CLUSTER_10.html]

Details for gene set DOROSZKO\_U3054MG\_CLUSTER\_10[GSEA]

|  || Dataset | state43017 |
| Phenotype | NoPhenotypeAvailable |
| Upregulated in class | na\_neg |
| GeneSet | DOROSZKO\_U3054MG\_CLUSTER\_10 |
| Enrichment Score (ES) | -0.36856368 |
| Normalized Enrichment Score (NES) | -2.0345416 |
| Nominal p-value | 0.0 |
| FDR q-value | 0.011614201 |
| FWER p-Value | 0.036 |
Table: GSEA Results Summary

  

Fig 1: Enrichment plot: DOROSZKO\_U3054MG\_CLUSTER\_10      
 Profile of the Running ES Score & Positions of GeneSet Members on the Rank Ordered List

  

| PROBE | GENE SYMBOL | GENE\_TITLE | RANK IN GENE LIST | RANK METRIC SCORE | RUNNING ES | CORE ENRICHMENT || 1 | FST |  |  | 272 | 0.362 | -0.3172 | Yes |
| 2 | CDH4 |  |  | 288 | 0.356 | -0.2871 | Yes |
| 3 | BCAP29 |  |  | 308 | 0.343 | -0.2641 | Yes |
| 4 | PDGFA |  |  | 328 | 0.335 | -0.2423 | Yes |
| 5 | GRIA4 |  |  | 358 | 0.322 | -0.2359 | Yes |
| 6 | PNRC2 |  |  | 364 | 0.321 | -0.1971 | Yes |
| 7 | SLC35F1 |  |  | 386 | 0.312 | -0.1813 | Yes |
| 8 | FABP7 |  |  | 414 | 0.302 | -0.1750 | Yes |
| 9 | LPCAT2 |  |  | 418 | 0.300 | -0.1365 | Yes |
| 10 | LAMC1 |  |  | 438 | 0.295 | -0.1204 | Yes |
| 11 | CEP350 |  |  | 455 | 0.291 | -0.1008 | Yes |
| 12 | SEMA3A |  |  | 460 | 0.290 | -0.0650 | Yes |
| 13 | PRKACA |  |  | 467 | 0.289 | -0.0322 | Yes |
| 14 | PLOD2 |  |  | 474 | 0.286 | 0.0003 | Yes |
| 15 | MOXD1 |  |  | 489 | 0.283 | 0.0215 | Yes |
| 16 | ACYP1 |  |  | 551 | 0.273 | -0.0225 | Yes |
| 17 | MMP16 |  |  | 707 | -0.285 | -0.1921 | Yes |
| 18 | ID4 |  |  | 708 | -0.285 | -0.1516 | Yes |
| 19 | CTXN1 |  |  | 715 | -0.309 | -0.1159 | Yes |
| 20 | MIR4458HG |  |  | 732 | -0.384 | -0.0830 | Yes |
| 21 | SQLE |  |  | 735 | -0.405 | -0.0282 | Yes |
| 22 | HMGCS1 |  |  | 739 | -0.418 | 0.0271 | Yes |
Table: GSEA details [plain text format]

  

Fig 2: DOROSZKO\_U3054MG\_CLUSTER\_10: Random ES distribution      
 Gene set null distribution of ES for **DOROSZKO\_U3054MG\_CLUSTER\_10**
